# A Functional Indel Polymorphism Within MIR155HG Is Associated With Sudden Cardiac Death Risk in a Chinese Population

**DOI:** 10.3389/fcvm.2021.671168

**Published:** 2021-05-31

**Authors:** Qing Zhang, Huan Yu, Zhenzhen Yang, Lijuan Li, Yan He, Shaohua Zhu, Chengtao Li, Suhua Zhang, Bin Luo, Yuzhen Gao

**Affiliations:** ^1^Department of Forensic Medicine, Medical College of Soochow University, Suzhou, China; ^2^Department of Epidemiology, Medical College of Soochow University, Suzhou, China; ^3^Shanghai Key Laboratory of Forensic Medicine, Institute of Forensic Sciences, Ministry of Justice, Shanghai, China; ^4^Faculty of Forensic Medicine, Zhongshan School of Medicine, Sun Yat-Sen University, Guangzhou, China

**Keywords:** sudden cardiac death, microRNA-155, rs72014506, indel polymorphism, genetic susceptibility

## Abstract

Sudden cardiac death (SCD) is a devastating complication of multiple disease processes and has gradually became a major public health issue. miR-155 is one of the best characterized miRNAs and plays a critical role in several physiological and pathological process, including cardiovascular diseases. In this study, we systematically screened the whole region of miR-155 host gene (*MIR155HG*) and identified a 4-bp insertion/deletion variant (rs72014506) residing in the intron region of *MIR155HG* as the candidate polymorphism. The association of rs72014506 with SCD susceptibility was evaluated using 166 SCD cases and 830 healthy controls in a Chinese population. Logistic regression analysis suggested that the homozygote del/del genotype significantly decreased the risk of SCD [odds ratio (OR) = 0.29; 95% confidence interval (CI) = 0.12–0.74; *P*_trend_ = 0.0004]. Further genotype–expression association study using human myocardium tissue samples suggested that the deletion allele was intimately linked to lower the expression of both MIR155HG and mature miR155. Luciferase activity assay also revealed that the deletion allele of rs72014506 inhibited gene transcriptional activity. Finally, we performed electrophoretic mobility shift assay and verified the preferential binding affinity of the deletion allele with POU2F1 (POU domain class 2 transcription factor 1). Collectively, we have successfully identified a SCD risk conferring polymorphism in the *MIR155HG* gene and a likely biological mechanism for the decreased risk of SCD associated with the deletion allele. This novel variant may thus serve as a potential genetic marker for SCD diagnosis and prevention in natural populations, if validated by further studies with a larger sample size.

## Introduction

Sudden cardiac death (SCD) is a devastating complication of multiple disease processes and has gradually become a major public health issue (1, 2). As an important cause of mortality in the general population, the incidence of SCD in western countries was 50–100 per 100,000 person-years (3), compared with 40.7 per 100,000 person-years in China (4). The sudden death of presumably well young people is devastating for their family and may have a large psychological impact on their surrounding community. SCD occurs in a broad spectrum of cardiac substrate pathologies, including coronary atherosclerotic diseases primarily in the older segment of the population, as well as primary and heritable electric disorders happening to children and young adults (5, 6). However, SCD patients in the general population are often observed with negative autopsy, which appeal to a molecular autopsy analysis using common genetic markers (7). A single common variant may contain limited effect size, but a combination of these significant variants would present a clear clinical picture of SCD. For these reasons, much progress has been made in illuminating the genetic underpinning of predisposition to SCD. Genome-wide association studies have revealed robust correlations of common genetic variants with SCD or SCD-related cardiac electric traits (8–10). Furthermore, the next-generation sequencing makes it possible to uncover genes involved in SCD at the multigene level (11, 12). The focus on germline variants could help to clarify SCD molecular mechanisms and therefore provide new insights into SCD prevention.

MicroRNAs represent a class of small non-protein-coding molecules involved in post-transcriptional gene regulation (13). They bind to 3′-untranslated regions (3′UTR) of mRNA and form a miRNA–mRNA silencing complex to repress the translation by mRNA degradation (13, 14). As one of the best characterized miRNAs, miR-155 is encoded by miR155 host gene (*MIR155HG*) located on chromosome 21. Emerging studies revealed the crucial role of miR-155 in several physiological and pathological process, including inflammation, immunity, and cardiovascular diseases (15, 16). For example, it has been shown that miR-155 had a great impact on determining macrophage phenotype and might be responsible for inflammation in atherosclerosis development (17, 18). MiR-155 was also found to be upregulated and was able to induce cardiac infiltration by macrophages and T lymphocytes during viral myocarditis, aggravating myocardial damage and deteriorating cardiac function (19). Genetic alteration in MiR-155 was also involved in cardiac pathology as previously reported. For example, previous studies have revealed that a mutation occurring within miR-155 target sites influenced AT1R protein expression and thereby contribute to susceptibility to hypertension (20, 21). However, contributions of *MIR155HG* variants to cardiovascular diseases still need to be investigated. Considering that miR-155 emerges as a key effector in cardiovascular biology, we hypothesized that *MIR155HG* variants may be associated with SCD occurrence.

In this study, we screened the whole region of *MIR155HG* and identified a 4-bp insertion/deletion(indel) variant (rs72014506) within the *MIR155HG* intron region. The case–control study was performed to evaluate the association between rs72016506 and SCD susceptibility in a Chinese population. Additional experiments were conducted to uncover the potential mechanisms underlying the association.

## Materials and Methods

### Ethics Statement

Our research was approved by the Ethical Committee of Soochow University (approval number: ME81772029). Informed consent was obtained from the relative of each victim before recruitment.

### Study Populations

A total of 166 SCD samples and 830 controls were enrolled in this research, and all the donors were genetically unrelated ethnic Han Chinese. The SCD blood samples were obtained from the following institutions during 2012–2019: Soochow University, Institute of Forensic Science, Ministry of Justice, and Sun Yat-sen University. The criterion of sample selection for the current study follows the principles described previously (22, 23). Briefly, all SCD cases caused by coronary heart diseases or hypertrophic cardiomyopathy were confirmed by rigorous forensic pathological investigation. No lethal pathological features were observed except for various degrees of coronary atherosclerosis. Moreover, forensic toxicological examinations in all cases ruled out the possibility of poisoning impact. Healthy controls with matched age (±5 years) and gender to SCD cases were enrolled from the community nutritional survey in the same areas and during the same period as the recruitment of the victims. These healthy donors were excluded from the control group, if they had any cardiovascular disease or family history of sudden death. Medicolegal Expertise Center of Soochow University provided additional 19 human myocardium tissues. The tissues were harvested at the time of forensic autopsy from traffic accident victims who were all healthy individuals. As soon as obtained from medicolegal autopsy, fresh heart tissues remained frozen at −80°C until the extraction of DNA and RNA.

### Screening of *MIR155HG* Variants

Information on all variants including SNPs and indels of *MIR155HG* was obtained from variation module in NCBI dbSNP database (https://www.ncbi.nlm.nih.gov/snp/). Due to the evolutionary disadvantage of uncommon risk alleles, a minimal minor allele frequency (MAF) > 0.1 was set up for variant selection strategy. Functional prediction to obtain regulative variants was achieved by searching the encyclopedia of DNA elements (ENCODE) database (24–26). Expression Quantitative Trait Loci (eQTL) analysis was performed in R (version 3.6.3) by pooling the target genotype data from the Ensembl database (http://www.ensembl.org/) with transcriptome data of 445 lymphoblastoid cell lines from 1000 Genome Project (27, 28).

### DNA Extraction and Genotyping

TIANamp Blood Spots DNA Kit or TIANamp Genomic DNA Kit (TIANGEN) was used for DNA extraction from blood samples, tissues, and cell lines. A pair of genotyping primers (see Supplementary Table 1) synthesized by Genewiz Company (Suzhou, China) was used for amplification of the polymorphic region. The PCR products were analyzed by 7% non-denaturing polyacrylamide gel electrophoresis and silver staining method (29). Genotyping results were conducted as described previously in a double-blinded way (30). As for quality control, we selected 50 random DNA samples for direct sequencing in order to validate the genotyping results. Approximately 10% of masked random samples were examined in blind duplicates by independent investigators to confirm a 100% concordance.

### RNA Extraction and Quantitative Real-Time PCR (qRT-PCR) Analysis

Total RNA of human myocardium tissue samples was collected with TRIzol (Invitrogen). Approximately 5 μg of RNA was used to synthesize first-strand cDNA and perform reverse transcription through Revert Aid First Strand cDNA Synthesis Kit (Cat #K1622, Thermo Scientific). Specific cDNA was generated using Bulge-Loop™ RT primers (Guangzhou RiboBio Co., Ltd.). SYBR® real-time PCR was performed on the Roche Light Cycler 96 system to quantify the relative expression level of MIR155HG and mature miR-155 in these samples, which was ultimately calculated by the 2^−Δ*ΔCT*^ algorithm. GAPDH or U6 small nuclear RNA (RNU6B) was chosen as the internal control. All the primer sequences used are listed in Supplementary Table 1.

### Cell Cultures

The 293T and HEC1B cell lines were cultured in Dulbecco's Modified Eagle Medium (DMEM) and Modified Eagle Medium (MEM), respectively. Both cells were supplemented with 10% fetal bovine serum (FBS) and 1% penicillin–streptomycin at 37°C in a humidified chamber supplemented with 5% CO_2_. These two cell lines were originated from Shanghai Cell Bank of Chinese Academy of Sciences and authorized by DNA analysis using short tandem repeat markers.

### Construction of Reporter Plasmid Vector and Luciferase Reporter Assay

DNA fragments containing rs72014506 (365 or 369 base pairs centered on the polymorphic site) were directly synthesized and subcloned into *Xho*I and *Hind*III sites of pGL3-control vector (Cat # E1741, Promega), obtaining the wild-type vector containing the insertion allele (pGL3-WT) and the mutant type vector (pGL3-MT) harboring deletion allele. To further examine whether the fragment containing different alleles has different promoter-driving ability, the *MIR155HG* promoter fragments (2 kb upstream of the transcription start site) were subcloned into *Kpn*I and *Nhe*I sites of pGL3-Basic vector (Cat # E1751, Promega), and the same restricted enzyme sites (*Xho*I and *Hind*III) were used to subcloned DNA fragments containing the indel polymorphism, named pGL3-proWT and pGL3-proMT, respectively.

A density of 1 × 10^5^ cells was seeded in one well of a 24-well-plate (Cat # 3524, Corning) for 24 h and then co-transfected with ~450 ng of reconstructed vector and 120 ng of pRL-TK vector (Promega) using Lipofectamine 2000 (Cat#11668-019, Invitrogen). The cells were lysed at 24 h post-transfection, and cell lysate was used to measure firefly luciferase activity by means of the Dual-Luciferase assay system (Cat #E1910, Promega), which were normalized with the Renilla luciferase activity. Each group was replicated in six wells, and each experiment was duplicated at least three times.

### Prediction of Transcription Factors

JASPAR (http://jaspar.genereg.net/) and TFBIND (http://tfbind.hgc.jp) were used to predict and analyze the binding of transcription factors at polymorphic sites (31, 32). Prediction results were exhibited by the UCSC genome browser. The correlation between transcription factor and *MIR155HG* gene expression level was obtained by Gene Expression Profiling Interactive Analysis (GEPIA); the expression data were from the Genotype-Tissue Expression (GTEx) database (33).

### Electrophoretic Mobility Shift Assays

Nuclear proteins from 293T were isolated by NE-PERTM Nuclear and Cytoplasmic Extraction Reagents (Cat# 78833, Thermo Scientific). The biotin probe labeled at both ends and non-labeled probe were synthesized by Viagene (Changzhou, China). The sequence of double-stranded oligonucleotides containing rs72014506 insertion or deletion alleles was listed in Supplementary Table 1. Nuclear protein extracts and biotin-labeled oligonucleotides were incubated at room temperature using the LightShift™ Chemiluminescent EMSA Kit (Cat# 20148X, Thermo Fisher), and detailed operations followed the kit protocol. For super-shift assays, the antibody against POU domain class 2 transcription factor 1 (POU2F1) (Cat #8157S, CST) was used. Reaction mixtures were separated by 6.5% native-polyacrylamide gel in 0.5 × Tris–boric acid buffer, and products were detected by the ECL system (ChemiScope, Shanghai).

### Statistical Analysis

Hardy–Weinberg equilibrium for control samples was assessed using chi-square testing. Unconditional logistic regression was used to analyze the associations between rs72014506 and SCD risk, adjusted by age and sex. The comparison of mean between two groups was assessed using the two-tailed Student's *t*-test. The Statistic Analysis System software (version 8.0, SAS Institute) and SPSS (version 20.0, SPSS Inc.) were implemented for analysis, and we set up *P* < 0.05 as the threshold of statistical significance. All statistical tests were two-sided in our study.

## Results

### Screening of Candidate Variants of *MIR155HG* Gene

**Figure 1:**
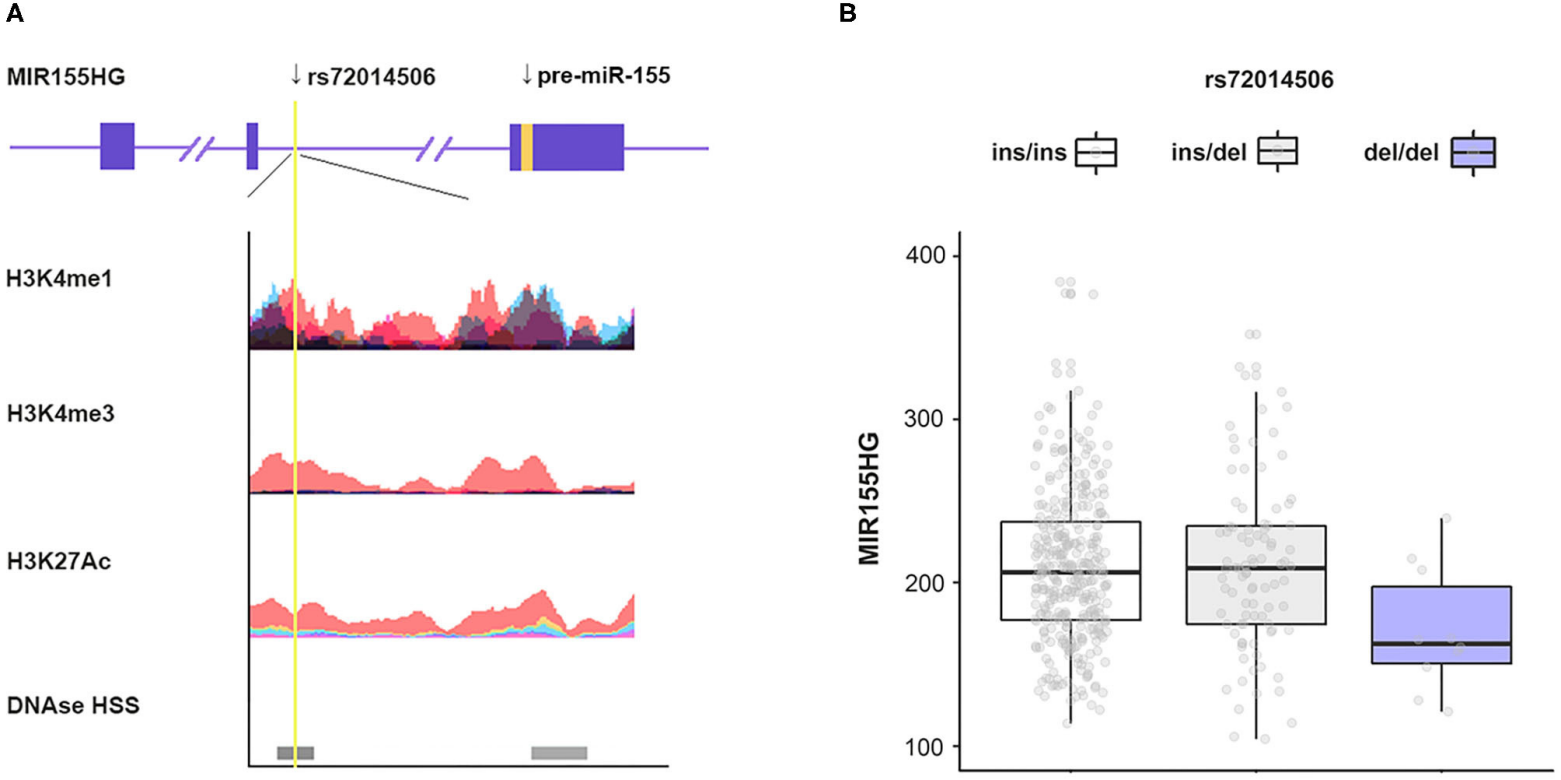
Screening of polymorphism. **(A)** Annotation of regions containing rs72014506 using the ENCODE database. The yellow line indicates the location of rs72014506. Colored histograms denote histone modification markers (H3K4me1, H3K4me3, and H3K27ac) in seven human cell types (GM12878, H1-hESC, HSMM, HUVEC, K562, NHEK, and NHLF). The darkness of DNase I hypersensitive represents the maximum signal strength observed in 95 cell lines. **(B)** MIR155HG mRNA expression levels as a function of genotype of rs72014506 determined by eQTLs analysis (*P* < 0.05).

Considering the wide application of capillary gel electrophoresis (CE) combined with PCR-based assays in forensic genetics, genetic marker selection priority was given to those length polymorphisms such as indels. Based on the specific screening strategy, only two indels (rs11295898 and rs72014506) were selected meeting the criterion in the whole region of *MIR155HG*. Additionally, a functional analysis based on ENCODE database was performed. The annotation by database suggested that the variant rs72014506 is rich in H3K4me1 or H3K4me3, and hypersensitive to DNaseI (Figure 1A). We then performed an eQTL analysis to screen variants associated with MIR155HG expression level; only rs72014506 presented a significant expression difference (Figure 1B). Thus, rs72014506 was chosen as the candidate variant for the current case–control study.

### The Associations of rs72014506 With SCD Susceptibility

**Figure 2:**
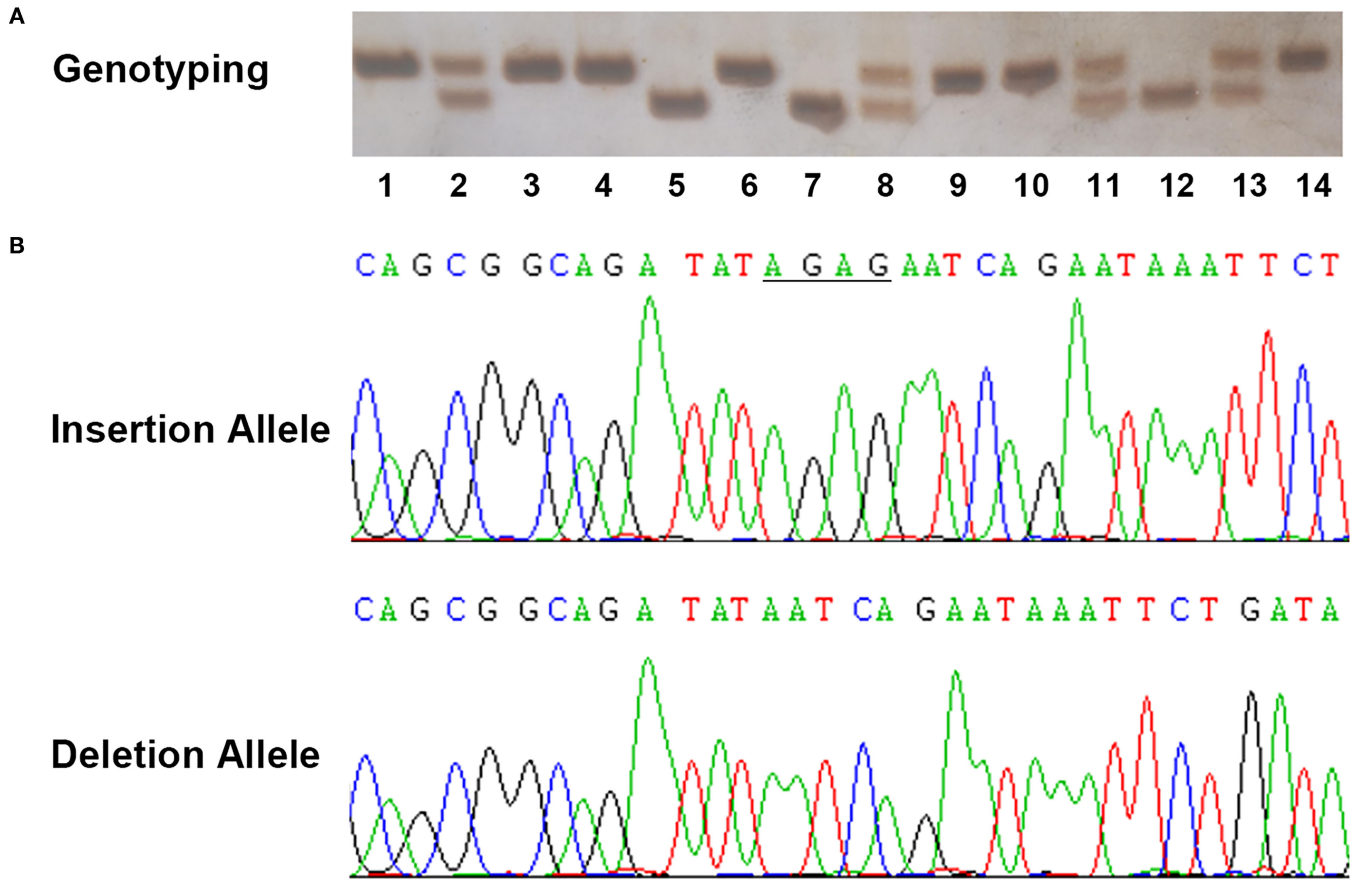
Example output from sequencing and genotyping assays of rs72014506. **(A)** The genotyping outcomes by using 7% non-denaturing polyacrylamide gel electrophoresis (PAGE) and silver staining (lanes 1, 3, 4, 6, 9, 10, and 14, ins/ins genotype; lanes 2, 8, 11, and 13, ins/del genotype; lanes 5, 7, and 12, del/del genotype). **(B)** The sequencing results of insertion and deletion allele in template strands. The underlined bases indicate the “AGAG” insertion in coding strands.

The demographic characteristics of both SCD cases and the healthy controls enrolled in the present study are summarized in Table 1. The detailed information for both SCD cases and controls are described in Supplementary Tables 2, 3. The average age of death for total SCD samples was 51.10 years old. As expected, SCD was more common in males, with a 10.07:1 ratio of male to female. Among all SCD cases, 37 cases (22.29%) happened after strenuous exercise or substantial physical activities; 51 cases (30.72%) occurred under stress conditions such as drastic emotional change, surgical treatment, and change of humoral homeostasis, which were classified as stress stimuli; 13 cases (7.83%) occurred while sleeping; 65 cases (39.16%) took place in a calm state or without witness, which were defined as non-specific. As megalothymus (hypertrophic thymus) had been previously linked to sudden infant death syndrome, the thymus status of SCD cases were examined during autopsy and only two cases suffered megalothymus. Examples of genotyping assay and sequencing results for rs72014506 were presented in Figure 2. The rs72014506 genotype distribution in the control group was in agreement with Hardy–Weinberg equilibrium (*P* > 0.05).

**Table 1:** Clinical characteristics of SCD cases and controls.

**Characteristics**	**Group**	
	**Case**	**Control**
No. of individuals	166	830
**Sex**, ***N***		
Male	151	756
Female	15	74
**Age, mean** **±** **SD (range)**		
Overall	51.10 ± 13.98 (19–92)	50.99 ± 13.83 (16–96)
Males	49.99 ± 12.89 (19–87)	49.93 ± 12.88 (16–86)
Females	62.33 ± 19.36 (27–92)	61.84 ± 18.08 (27–96)
**BMI, mean** **±** **SD (range)**		
Overall	23.97 ± 2.89 (17.28–32.57)	23.95 ± 2.87 (16.56–32.99)
Males	24.00 ± 2.93 (17.28–32.57)	23.97 ± 2.92 (16.56–32.99)
Females	23.71 ± 2.49 (20.35–27.85)	23.72 ± 2.36 (19.18–27.56)
**Events at sudden death (SD)**		
Physical activity	37	
Stress	51	
Sleep	13	
Non- specific	65	
**Symptoms before SD**		
None	116	
Others	50	
**Megalothymus**		
Positive	2	
Negative	164	

Genotypic frequencies of rs72014506 and odds ratio (OR) with its 95% confidence interval (CI) are shown in Table 2. Compared with rs72014506 ins/ins genotype carriers, del/del carriers had a lower SCD risk [OR = 0.29, 95% CI (0.12–0.74), *P* = 0.0058]. At the allelic level as shown in the additive model, the deletion allele was associated with a significantly reduced risk of SCD [OR = 0.56, 95% CI (0.41–0.76), *P* = 0.0002]. Collectively, these results suggest the significant correlations of rs72014506 with SCD susceptibility in a Chinese population. Additionally, we provided some postmortem autopsy findings such as heart weight and ventricular thickness, but no significant correlation was observed after risk stratification analysis.

**Table 2:** Associations between rs72014506 and sudden cardiac death susceptibility in case–control sets recruited during 2012–2019.

**Genetic Model**	**Genotype**	**Cases**	**(%)**	**Control**	**(%)**	**OR (95% CI)^a^**	***P-*value**
Codominant model	ins/ins	116	69.88	465	56.02	1.00 (Reference)	
	ins/del	45	27.11	296	35.66	0.61 (0.42–0.87)	0.0090
	del/del	5	3.01	69	8.32	0.29 (0.12–0.74)^*^	0.0058
	*P*_trend_						0.0004
Dominant model	ins/ins	116	69.88	465	56.02	1.00 (Reference)	
	ins/del+del/del	50	30.12	365	43.98	0.55 ( 0.38–0.79)	0.0009
Additive model	ins allele	277	83.43	1,226	73.86	1.00 (Reference)	
	del allele	55	16.57	434	26.14	0.56 (0.41–0.76)	0.0002

a *Adjusted by age and sex. CI, confidence interval; OR, odds ratio*.

* *Fisher's exact test*.

### Genotype–Phenotype Analysis Between rs72014506 and Expression of MIR155HG and miR-155

**Figure 3:**
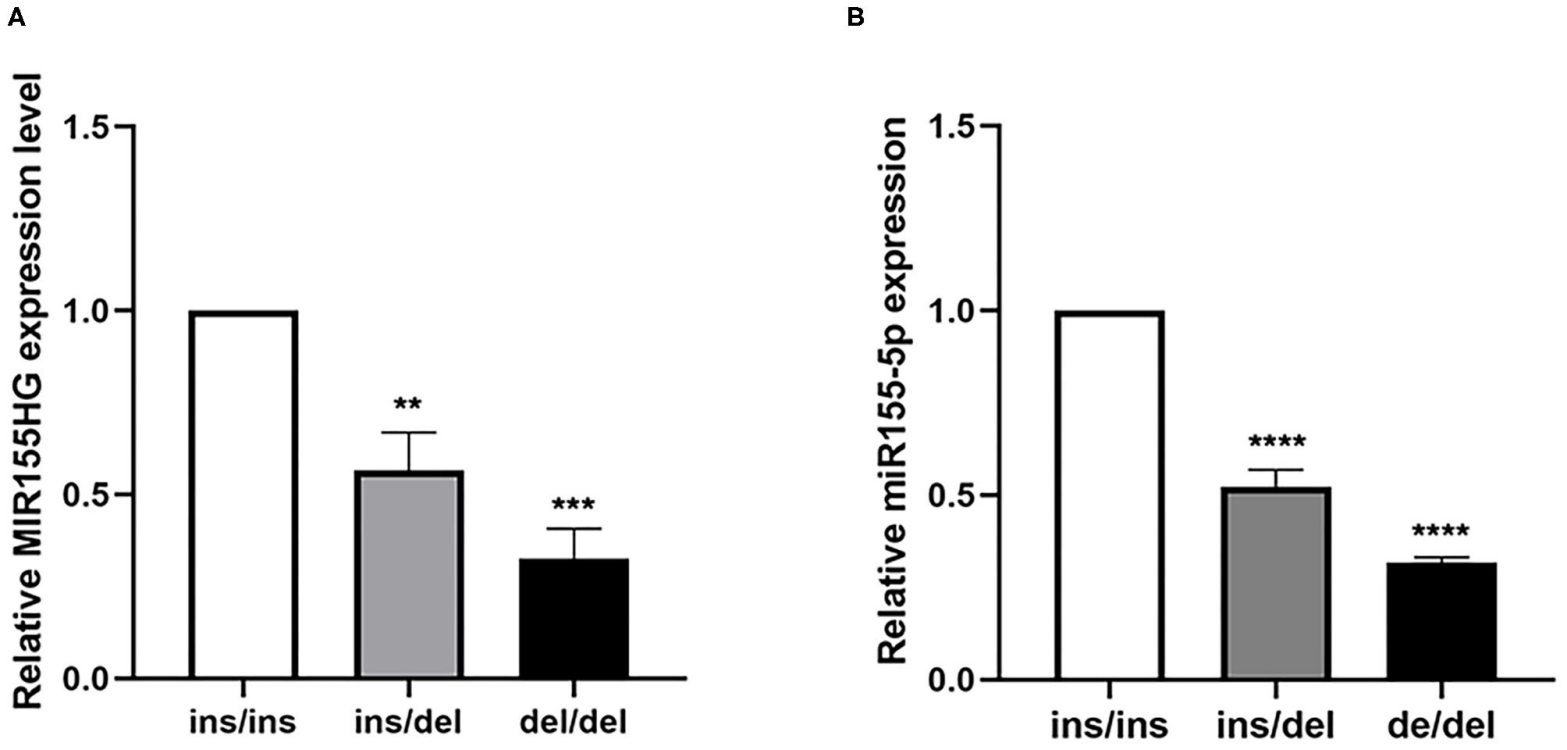
The expression levels of MIR155HG and mature miR-155 in human myocardium tissues with different genotypes. **(A)** The mRNA level of MIR155HG in human samples showed that in tissues with the ins/del and del/del genotype was 0.42- and 0.68-fold lower than that in samples with ins/ins genotype (**P < 0.01, ***P < 0.001, ins/ins, *N* = 9, ins/del, *N* = 5, del/del, *N* = 5). **(B)** Mature miR-155 expression in tissues showed the same tendency as MIR155HG. Myocardium samples with the ins/del and del/del genotype was 0.48- and 0.68-fold lower than that with ins/ins samples, respectively (****P < 0.0001, ins/ins, *N* = 9, ins/del, *N* = 5, del/del, *N* = 5).

We examined the expression levels in human myocardium tissue samples with different genotypes to further investigate the effect of the polymorphism on the expression levels of MIR155HG and mature miR-155. As shown in Figure 3A, the MIR155HG expression level in samples with the ins/del and del/del genotype appeared to be significantly lower than that in samples with ins/ins genotype. Similarly, the mature miR-155 expression level of samples with the ins/del and del/del genotype was 0.48- and 0.68-fold lower than that with ins/ins-genotype samples (Figure 3B). These results thus suggested that the deletion allele of rs72014506 was associated with decreased MIR155HG/MIR-155 transcription.

### The Influence of rs72014506 on Gene Transcription Activity

**Figure 4:**
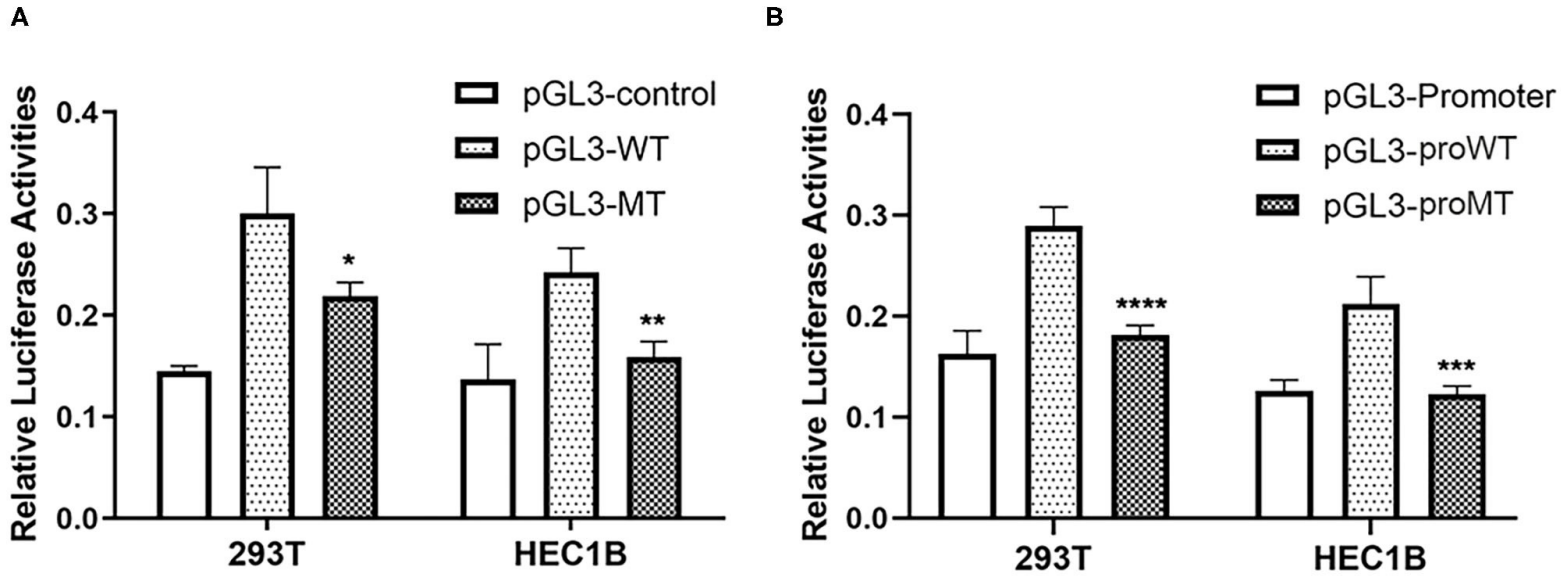
The effect of rs72014506 on gene transcriptional activity. **(A)** The relative firefly luciferase activities were compared between insertion construct group (pGL3-WT) and deletion construct group (pGL3-MT) in 293T and HEC1B cell lines. Cells transfected with pGL3-MT exhibited a significantly lower luciferase activity as compared with cells transfected with pGL3-WT (*P < 0.05, **P < 0.05). **(B)** Relative reporter gene expression in constructs containing *MIR155HG* promoter driven by DNA fragment harboring rs72014506 in 293T and HEC1B (pGL3-proWT, insertion allele; pGL3-proMT, deletion allele). Cell group transfected with constructs containing rs72014506 deletion allele showed lower firefly luciferase activities than that harboring insertion allele vectors (***P < 0.001, ****P < 0.0001).

In order to investigate the effect of rs72014506 on the transcriptional activity of MIR155HG, we created promoter luciferase constructs containing insertion or deletion alleles and dual luciferase assay was carried out in 293T and HEC1B cell lines that contained ins/ins and del/del genotypes, respectively. As shown in Figure 4A, there was a significant difference in firefly luciferase expression between cell lines transfected by two allele-different fragments. The group transfected with pGL3-WT presented higher luciferase activity than that of the group transfected with pGL3-MT, indicating that the deletion DNA fragments may have a regulatory function. Given that rs72014506 resides in the second intron of *MIR155HG*, it might repress the gene transcription activity through a long-range interaction with the promotor. Remarkably, the group transfected with the vector containing the *MIR155HG* promoter region further confirmed our hypothesis. The group transfected with pGL3-proMT displayed a lower luciferase activity than that the pGL3-proWT transfection group (Figure 4B). The results suggested the influence of rs72014506 on the *MIR155HG* promoter and its possible regulatory role on gene transcription.

### Repression of MIR155HG/miR-155 Expression by rs72014506 Is Mediated by POU2F1

**Figure 5:**
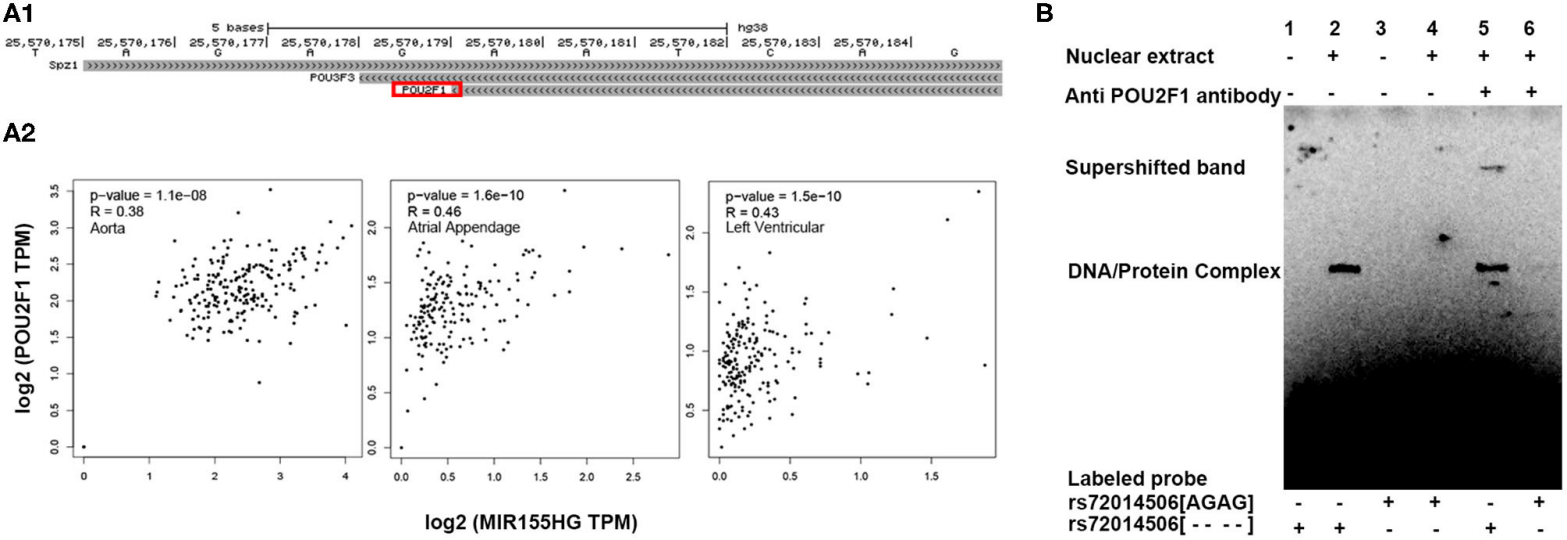
POU2F1 mediated the interaction of rs72014506 with MIR155HG expression. **(A)** Transcription factor prediction and correlation analysis results. **(A1)** This UCSC Genome Browser track hub represents genome-wide predicted binding sites for transcription factor binding profiles in the JASPAR database CORE collection; this view only showed predicted binding sites with scores above 350, which indicated that the *P*-value of the TF match to indel position was <0.001. The same TFs in TFBIND prediction result were highlighted by a red square. **(A2)** The expression of POU2F1 and MIR155HG presents a significant correlation in human aorta, atrial appendage, and left ventricular tissues. **(B)** Electrophoretic mobility shift assays with biotin-labeled DNA sequence containing the rs72014506[AGAG] or rs72014506 [- - - -] and nuclear extracts from 293T cells. Lanes 1 and 3 show mobility of the labeled oligonucleotides without nuclear extracts; lanes 2 and 4 show mobility of the labeled oligonucleotides with nuclear extracts. A DNA–protein complex was presented when [- - - -]-containing DNA was incubated with nuclear extracts (lane 2) but not [AGAG]-containing probe (Lane 4). In super-shift EMSA, the super-shifted band only appeared when anti-POU2F1 antibody was incubated with rs72014506[- - - -]-labeled probe (Lane 5).

We hypothesized that the deletion variant of rs72014506 might repress MIR155/miR-155 expression by recruiting transcriptional inhibitors. *In silico* analysis suggested that the rs72014506 deletion allele may create a binding site for transcription factor POU2F1 in this region (Figure 5A1). Gene correlation analysis also presented a high relationship between MIR155HG and POU2F1 in different kinds of human tissues (Figure 5A2). Therefore, we performed electrophoretic mobility shift assays to verify the conjecture and found that the binding patterns between the rs72014506[AGAG] and rs72014506[- - - -] biotin-labeled probes were indeed different. A DNA–protein complex explicitly appeared when the rs72014506[- - - -]- but not the [AGAG]-containing probe was incubated with nuclear protein extracts (Figure 5B, lane 2). Furthermore, when the specific antibody against POU2F1 was incubated with the DNA–protein mixture, the original binding band was shallower and shifted upward and a super-shift band can be observed (lane 5), which suggested that the rs72014506[AGAG] to rs72014506[- - - -] change may create a binding site to transcription factor POU2F1.

## Discussion

Coronary atherosclerosis has long been recognized to be associated with SCD (34). Given that miR-155 is an important determinant of pathologies to atherosclerosis, mutations within *MIR155HG* may play a critical role in SCD. In this study, we initially identified that one 4-bp indel polymorphism (rs72014506) in the intron region of *MIR155HG* was functionally relevant to SCD susceptibility in a Chinese population. Moreover, our functional studies demonstrated that the deletion allele of rs72014506 inhibited the expression of both MIR155HG and mature miR-155 through an allele-dependent manner. Finally, we uncovered that the rs72014506[AGAG] to rs72014506[- - - -] change may create a POU2F1 binding site. These results provided a plausible mechanism underlying the decreased risk of SCD, which associated with rs72014506[- - - -], and the rs72014506 polymorphism might serve as a potential genetic marker for SCD diagnosis and prevention.

Inflammation contributes to the earliest lesions and acts as a central component of atherosclerosis (35, 36). High level of low-density lipoprotein (LDL) induces the production of free radicals and was thereby transformed into oxidized LDL (oxLDL). The endothelium injured by oxLDL releases more adhesion molecules, which contributes to the adherence and accumulation of monocytes and T cells. Macrophages derived from monocytes internalize the oxLDL and finally lead to the formation of foam cells and atherosclerotic plaques. Furthermore, a large number of monocytes and macrophages aggregated by inflammation would secrete matrix metalloproteinases and erosion the fibrous cap, making the plaques more vulnerable. These inflammatory cells also interact with platelets and continuously promote the recruitments of platelets and inflammatory cells, maintaining the acute coronary syndrome and promoting thrombosis. At last, rupture of coronary plaques would occur, leading to acute myocardial ischemia and sudden death (37).

MiR-155 is one of key regulators of inflammation, but the effects of miR-155 on inflammation and atherosclerosis are conflicting. It has been shown that miR-155-5p delivered from vascular adventitial fibroblasts could repress the oxidative stress and inflammation in vascular smooth muscle cells (VSMCs) (38). MiR-155 was also reported to attenuate the formation of foam cells in coronary atherosclerosis and relieves the chronic inflammation by inhibiting calcium-regulated heat stable protein 1 (CARHSP1) and tumor necrosis factor alpha (TNF-α) (39). These studies suggested that miR-155 acted as an anti-inflammatory factor, while mounting evidences have also indicated its pro-inflammatory roles. MiR-155 has been demonstrated to determine macrophage phenotype by the linkage to M1 pro-inflammatory macrophage (16). Furthermore, miR-155 delivered by neutrophil micro-vesicles could enhance the NF-κB expression of endothelial cells in atheroprone regions, thereby aggravating macrophage infiltration and plaque formation (18). These researches indicated that miR-155 in different cells may play different roles in inflammatory processes. In various atherosclerotic animal models, miR-155 suppression also leads to contradictory effects. The miR-155 deficiency had reduced macrophage inflammation and attenuated atherosclerotic lesion in ApoE^−/−^ mice, while Ldlr^−/−^ and miR-155^−/−^ mice appeared to elevate levels of monocytes and more severe atherosclerotic plaques (40, 41). Given that ApoE^−/−^ and Ldlr^−/−^ mice reflect different stages of atherosclerosis, miR-155 may also play a stage-specific role. Overall, miR-155 plays a key role in macrophage inflammation during atherosclerotic development and therefore may prevent plaque rupture. In our study, most of the included SCD cases suffered varying degrees of atherosclerosis. Our study also uncovered that rs72014506 del allele is the protective allele for SCD and is intimately linked to the miR-155 suppression. Considering the role of miR-155 deficiency in ApoE^−/−^ mice that resulted in advanced atherosclerosis, we therefore hypothesized that this miR-155 inhibition induced by the genetic variant could reduce macrophage migration and atherosclerosis progression and therefore decreased the risk of plaque rupture during advanced atherosclerosis and prevented patients from SCD.

Non-coding mutations are always reported to associate with disease phenotype by interacting with gene promoter through a long-range mechanism (42–44). These mutants would create an enhancer or silencer and a binding site to transcription factor, thereby regulating transcription of key genes involved in disease development. POU2F1, a transcription factor also known as OCT1, has been demonstrated to interact with allele rs72014506[- - - -] in our study. It has been previously reported that POU2F1 was able to bind to upstream of interleukin-5 (IL-5) and suppressed IL-5 expression in T cells (45), while it served as a transcription activator for lectin-like oxLDL receptor 1 (LOX-1), an important atherogenic trigger (46). In the present study, our bioinformatic analysis revealed that rs72014506 locus is rich in H3K4me1 or H3K4me3, and hypersensitive to DNaseI, which all indicated that rs72014506 may locate in a cis-regulatory element. Our observations by functional experiments further suggested that rs72014506[- - - -] downregulated the expression of MIR155HG and miR-155. Considering that the rs72014506[AGAG] to rs72014506[- - - -] change may create a binding site to POU2F1, the binding of POU2F1 might be correlated with the repression of MIR155HG/miR-155, which may finally decrease the risk of SCD.

It is well-known that SCD caused by coronary artery disease (CAD) accounts for the largest proportion of all SCD patients. All the victims in our research suffered various degrees of coronary atherosclerosis and were diagnosed as sudden coronary atherosclerotic death by forensic autopsy. The case–control study has observed a positive association between rs72014506 and SCD risk, which raise the possibility that this indel may be associated with coronary atherosclerosis susceptibility in natural population, and thus serve as a molecular diagnostic marker for coronary atherosclerotic. However, this speculation still needs to be further explored by CAD-related studies, which may have potential interests for cardiovascular community. Another limitation is the insufficient clinical characteristics of SCD cases. The enrollment and analysis of the SCD cases in the current study were performed in a forensic context. Majority of the SCD samples were collected from those out-of-hospital sudden death cases without any emergency medical services. Therefore, the detailed clinical information of SCD cases was very limited. On the other hand, the record of cardiovascular comorbidities such as hypertension and diabetes was not taken into consideration in the current study since the information was mostly derived from relatives' self-report without any verification. This could not preclude under-reporting of comorbidity and limit the ability to determine causal pathways between putative risk factors and SCD. For these reasons, the transmission of our observations in the clinical setting still needs further validation studies with better propensity score matching.

## Conclusion

Using a candidate-gene-based genetic association study, we have successfully identified a risk conferring indel polymorphism in the *MIR155HG* gene and a likely biological mechanism for decreased risk of SCD associated with the deletion allele. This novel variant may thus serve as a potential genetic marker for SCD diagnosis and prevention in natural populations, if validated by further studies with a larger sample size.

## Data Availability Statement

The original contributions presented in the study are included in the article/Supplementary Material, further inquiries can be directed to the corresponding author/s.

## Ethics Statement

The studies involving human participants were reviewed and approved by Ethical Committee of Soochow University (approval number: ME81772029). Written informed consent to participate in this study was provided by the participants' legal guardian/next of kin.

## Author Contributions

YG and BL: conceived and designed the experiments. QZ, HY, and ZY: performed the experiments. LL and YH: analyzed the data. SZhu, CL, and SZha: contributed reagents/materials/analysis tools. QZ, HY, and YG: contributed to the writing of the manuscript. QZ and YG: contributed to manuscript editing. All authors contributed to the article and approved the submitted version.

## Conflict of Interest

The authors declare that the research was conducted in the absence of any commercial or financial relationships that could be construed as a potential conflict of interest.
