# Supplementary material for: A Functional Indel Polymorphism Within MIR155HG Is Associated With Sudden Cardiac Death Risk in a Chinese Population
Source: Front Cardiovasc Med. 2021 May 31;8:671168. doi: 10.3389/fcvm.2021.671168 (PMC8200405; doi:10.3389/fcvm.2021.671168)
Supplement: Supplementary file 1 [file Table_1.DOCX]

**Supplemental table 1. Sequences**

1. **Genotyping primers sequence**

| Primer | Sequence |
| --- | --- |
| Forward primer | 5’- CACTTGCATTAATCACTGCT -3’ |
| Reverse primer | 5’- GTATATGTGGAAACAGTATCTGC -3’ |

1. **Primer sequences of quantitative real-time PCR analysis.**

| Primer | Sequence |
| --- | --- |
| MIR155HG-F | 5′- GTGGCACAAACCAGGAAGG -3′ |
| MIR155HG-R | 5′- AGTTGGAGGCAAAAACCCCT -3′ |
| GAPDH-F | 5′- CTCTCTGCTCCTCCTGTTCGAC-3′ |
| GAPDH-R | 5′- TGAGCGATGTGGCTCGGCT -3′ |

1. **Biotin-labeled double-stranded oligonucleotides sequences**

| Probe | Sequence |
| --- | --- |
| WT-TP | 5’biotin-CGGCAGAATATAGAGAATCAGAATA-3’ biotin |
| WT-BM | 5’biotin-TATTCTGATTCTCTATATTCTGCCG -3’ biotin |
| MT-TP | 5’biotin- CGGCAGAATATAATCAGAATA -3’ biotin |
| MT-BM | 5’biotin- TATTCTGATTATATTCTGCCG -3’ biotin |

WT, insertion allele; MT, deletion allele. Sequences of unlabeled probes were same with biotin-labeled probes.
